# Manganese-Based Prussian Blue Nanocatalysts Suppress Non-Small Cell Lung Cancer Growth and Metastasis *via* Photothermal and Chemodynamic Therapy

**DOI:** 10.3389/fbioe.2022.939158

**Published:** 2022-06-22

**Authors:** Danruo Fang, Zeyu Liu, Hansong Jin, Xiulin Huang, Yongxin Shi, Suqin Ben

**Affiliations:** ^1^ Department of Respiratory and Critical Care Medicine, Shanghai General Hospital, Shanghai Jiao Tong University School of Medicine, Shanghai, China; ^2^ Department of Respiratory and Critical Care Medicine, Shanghai Jiao Tong University Affiliated Sixth People’s Hospital, Shanghai, China; ^3^ Department of Thoracic Surgery, Shanghai General Hospital, Shanghai Jiao Tong University School of Medicine, Shanghai, China

**Keywords:** MnPB nanoparticles, non-small cell lung cancer, chemodynamic therapy, photothermal therapy, metastatic lung cancer

## Abstract

Based on the safety of prussian blue (PB) in biomedical application, we prepared manganese-based prussian blue (MnPB) nanocatalysts to achieve enhanced photothermal therapy and chemodynamic therapy. And we conducted a series of experiments to explore the therapeutic effects of MnPB nanoparticles (NPs) on non-small cell lung cancer (NSCLC) *in vivo* and *in vitro*. For *in vitro* experiments, the MnPB NPs suppressed growth of A549 cells by reactive oxygen species upregulation and near-infrared irradiation. Moreover, the MnPB NPs could inhibit lung cancer metastasis through downregulating the matrix metalloproteinase (MMP)-2 and MMP-9 expression in A549 cells. And for *in vivo* experiments, the MnPB NPs inhibited the growth of xenografted tumor effectively and were biologically safe. Meanwhile, Mn^2+^ as a T1-weighted agent could realize magnetic resonance imaging-guided diagnosis and treatment. To sum up, the results in this study clearly demonstrated that the MnPB NPs had remarkable effects for inhibiting the growth and metastasis of NSCLC and might serve as a promising multifunctional nanoplatform for NSCLC treatment.

## Introduction

Lung cancer is a common and severe global health problem, as it has become the first and third major cause of cancer related mortality among males and females respectively (Altorki et al., 2019; Zheng et al., 2021). And non-small cell lung cancer (NSCLC) is the main histological subtype, accounting for about 85% (Oser et al., 2015). Although recent therapeutic advances in surgery, chemoradiotherapy and targeted therapy, a substantial proportion of patients (75%) diagnosed with metastatic lung cancer has poor 5-year relative survival rate (6%) (Siegel et al., 2021). Additionally, drug resistance, toxicity and limited efficacy of many therapeutic compounds are pivotal impediments to the successful treatment of NSCLC. Consequently, it is critical to develop other safe and effective treatments to combat this deadly disease.

Recently, with the rapid progress of nanomedicine, nanoparticle systems have attracted considerable attention for various applications of tumor diagnosis and treatment (Lim et al., 2015; Li J. et al., 2021). And some new therapeutic modalities, including photothermal therapy (PTT) and chemodynamic therapy (CDT), exhibit good effect on tumor treatment (Guan et al., 2021; Sun et al., 2021). PTT, as a promising non-invasive cancer treatment strategy, can convert photon energy into heat to eradicate tumor cells (Li et al., 2020; Zhao H. et al., 2021). And PTT exhibits several advantages, including limited side-effects, high spatial selectivity and minimal injury to normal tissues (Wang et al., 2020a; Zheng et al., 2020; He et al., 2021). Meanwhile, CDT is an emerging nanocatalyst-based cancer treatment, which decomposes hydrogen peroxide (H_2_O_2_) to generate toxic reactive oxygen species (ROS) by Fenton or Fenton-like reactions (Wang et al., 2020b; Yang et al., 2021). It is well known that ROS can induce protein, DNA, and lipid damage to cause tumor cell death (Goldstein et al., 1993; Lin et al., 2020). The combination of PTT and CDT has been drawing more and more research attention because of their high-efficacy (Manivasagan et al., 2022).

Prussian blue (PB), a mixed-valence iron (III) hexacyanoferrate (II), has been widely explored as a powerful tool in biomedical research due to some superior properties, including excellent biocompatibility, good biodegradability, easy synthesis and favorable thermal stability (Busquets and Estelrich, 2020; Gao et al., 2020). Besides, PB has been authorized by USA Food and Drug Administration (FDA) as a safe material for clinical applications (Odda et al., 2019). But PB nanoparticles (NPs) with low catalase-like activity and low photothermal conversion efficiency are not promising agents for PTT and CDT (Dacarro et al., 2018; Gao et al., 2020). Moreover, although PB NPs have been reported as T1-weighted contrast agents for magnetic resonance imaging (MRI) (Shokouhimehr et al., 2010), they have a weak effect on longitudinal relaxation (r1), resulting in the low diagnostic ability of tumor (Li et al., 2014). Since Mn^2+^, Zn^2+^ and Gd^3+^ have good performance on enhancing T1 weighted MRI, it is an effective strategy to add metal ions into PB NPs for improving r1 value (Cai et al., 2015; Shou et al., 2020; Zhao W. et al., 2021). The manganese-based prussian blue (MnPB) nanocatalysts fabricated in this study not only possess excellent CDT/PTT effect, but also show high longitudinal relaxivity. Mn^2+^ in MnPB NPs enhances catalase-like activity of PB and improves PTT function resulting from strengthening optical absorption or shifting absorption to the near-infrared (NIR) region. Therefore, MnPB NPs in this study have strong synergistic effects and may shed light on potential therapies for growth and metastatic of NSCLC.

Tumor metastasis is a complicated mechanism involving tumor cell adhesion, migration and the degradation of the extracellular matrix (ECM) (Paolillo and Schinelli, 2019). Matrix metalloproteinases (MMPs) are zinc-dependent endopeptidases, playing a critical role in degrading the ECM (Gonzalez-Avila et al., 2019). Among MMPs, MMP2 and MMP9 are important members of MMP family, involving in the invasion and metastasis of NSCLC by degrading basement membrane and matrix collagen (Dong et al., 2013; Wang et al., 2018). A large number of scientific studies have shown that MMP2 and MMP9 are highly expressed in lung cancer tissues (Li et al., 2019; Han et al., 2020). Furthermore, the downregulation of MMP2/MMP9 can significantly inhibit tumor cell proliferation, invasion and metastasis (Itoh et al., 1999; Poudel et al., 2016). We found that MnPB NPs synthesized in our study could significantly inhibit MMP-2/MMP-9 expression and might be promising therapeutic agents to suppress growth and metastasis of NSCLC.

In the current study, we fully explored the anti-NSCLC effect of novel MnPB nanocatalysts *in vitro* and *in vivo*. The experimental results showed that MnPB nanocatalysts could serve as effective MRI-guided agents for synergistic chemodynamic/photothermal therapy and inhibit the growth and metastasis of NSCLC effectively.

## Materials and Methods

### Materials

MnCl_2_·4H_2_O (AR, 99.0%) and K_4_[Fe(CN)_6_]·3H_2_O (≥99.99%, metals basis) were acquired from Macklin Biochemical Technology Co., Ltd. (Shanghai, China) and Aladdin Chemistry Co., Ltd. (Shanghai, China) respectively. Citric acid was acquired from Sinopharm Chemical Reagent Co., Ltd. (Shanghai, China). Absolute ethyl alcohol (C_2_H_5_OH; AR) was purchased from Sinopharm Chemical Reagent Co., Ltd. (Shanghai, China). The deionized water (H_2_O) was purified with a Milli-Q system (Millipore, Bedford, MA, United States). All chemicals and solvents were not further purified to use.

### Synthesis of Manganese-Based Prussian Blue Nanoparticles

MnPB NPs were creatively synthesized by a facile ion-exchange method on the grounds of the co-precipitation strategy. First, MnCl_2_·4H_2_O (0.3 mM) and citric acid (0.5 mM) dissolved in the deionized water (20 ml) were heated to 60°C for 5 min. The obtained mixture was served as solution A. Then, K_4_[Fe(CN)_6_] (0.4 mM) and citric acid (0.4 mM) were added together in the deionized water (20 ml) to become solution B. The solution B was also heated to 60°C for 5 min under magnetically stirring. Next, the solution B was added dropwise to the solution A. Then the mixed solution was magnetically stirred and kept at 60°C for 2 min. And the collected product was subsequently washed by deionized water and ethonal after the reaction was cooled down to room temperature. Finally, washed solution was then dried at 60 °C in a vacuum oven for 24 h.

### Characterization of Manganese-Based Prussian Blue Nanoparticles

The particle size and morphology of MnPB NPs were measured by a JEM-JEOL-200 transmission electron microscopy (TEM, Tokyo, Japan) operating at 200 kV. The hydrodynamic size of MnPB NPs was analyzed by dynamic light scattering (DLS, Brookhaven Instrument Corporation, United States). And we used X-ray powder diffraction (XRD, Rigaku, Japan) to reveal the crystal structure and functional group of MnPB NPs.

### Photothermal Properties of Manganese-Based Prussian Blue Nanoparticles

To evaluate the light absorption ability of MnPB NPs, the absorbance of MnPB NPs at various concentrations (100, 200 and 400 μg/ml) was measured *via* ultraviolet-visible-NIR (UV-vis-NIR) spectrophotometer (Agilent, CA, United States). Next, aqueous dispersion of MnPB NPs with different concentrations was exposed to irradiation with a NIR laser (808 nm, 1.0 W/cm^2^). During laser irradiation period, we used an infrared thermal imaging camera (Fotric, China) to record the temperature change. Next, 200 μg/ml solution of MnPB NPs was irradiated with the 808 nm NIR laser light (1.0 W/cm^2^) for another 10 min and cooled down to the room temperature to get temperature curves and photothermal conversion efficiency (η) of MnPB NPs. The formula of η is as follows:

$$ŋ=\frac{hS\left(T_{max}-T_{surr}\right)-Q_{Dis}}{I\left(1-10^{-A_{808}}\right)}$$

where h is the heat transfer coefficient, S is the surface area of the container, T_max_ is the maximum steady-state temperature of the sample solution, T_surr_ is the ambient surrounding temperature, Q_Dis_ is the heat input due to light absorption by the solvent and container, I is the laser power, and A_808_ is the absorbance of the sample solution at 808 nm (Liu X. et al., 2014).

### Determination of Reactive Oxygen Species Generation

The ROS generation tests were divided into three groups: (1) H_2_O_2_ (200 μM) solution; (2) MnPB (1 mg/ml) solution; (3) MnPB (1 mg/ml) + H_2_O_2_ (200 μM) solution. Then we added methylene blue (MB) (5 μg/ml) in two groups to detect the ROS generation. And the UV-vis-NIR absorption spectra of these solutions were scanned in time-scan mode.

### Cytotoxicity

BEAS-2B cells (normal human bronchial epithelial cells) and A549 cells (human lung adenocarcinoma cells) were inoculated into 96-well plates respectively and cultured for 24 h. Then the culture mediums were replaced by the fresh mediums with various concentrations of MnPB NPs. After 24 h of treatment, the mediums were detected by cell counting kit-8 (CCK-8) assay (Beyotime Biotechnology, China).

### *In Vitro* Reactive Oxygen Species Detection

A549 cells were cultured in the 6-well plates and categorized into four groups: the control group, the H_2_O_2_ (100 μM) group, the MnPB (400 μg/ml) group and the MnPB (400 μg/ml) + H_2_O_2_ (100 μM) group. After 24 h of treatment, intracellular ROS levels were assessed using the probe solution 2, 7-dichlorodihydrofluorescein diacetate (DCFH-DA, Beyotime Biotechnology, China). After incubated with DCFH-DA for 20 min, the cells were washed three times with PBS and replaced with serum-free mediums. Last, images were obtained under a fluorescence microscope (Leica DMi8, Leica, Germany).

### Calcine-AM/Propidium Iodide Test and Annexin V-FITC Apoptosis Assay

The percentage of living and dead cells of A549 cells was examined by Calcine-AM/propidium iodide (PI) test and the apoptosis of A549 cells was detected the Annexin V-FITC apoptosis kit. For the Calcine-AM/PI test (Beyotime Biotechnology, China), A549 cells were cultured in the complete medium at pH 6.5 and divided into six groups: 1) control; 2) NIR; 3) H_2_O_2_ (100 μM); 4) MnPB (400 μg/ml) + NIR; 5) MnPB (400 μg/ml) + H_2_O_2_ (100 μM); 6) MnPB (400 μg/ml) + H_2_O_2_ (100 μM) + NIR. After 12 h of corresponding treatments, the group 2, 4, and 6 were irradiated with 808 nm NIR (1.0 W/cm^2^) for 10 min. Then, cells were dyed with Calcein-AM and PI for 15 min. Finally, we acquired images using a fluorescence microscope (Leica DMi8, Leica, Germany).

In Annexin V-FITC apoptosis assay (Multi Sciences, China), the cell grouping and treatments were consistent with the Calcine-AM/PI experiment. A549 cells were collected and washed with cold PBS. Next, the cells were resuspended in 1 × binding buffer (500 μl) containing Annexin V-FITC (5 μl) and incubated for 5 min in the dark. And we utilized flow cytometry (cytoflex LX, Beckman Coulter) to assess the ratio of apoptotic cells.

### Transwell Migration Assay

We used 24-well Transwell chambers (Corning, United States) with a polycarbonate filter membrane of 8 μm pore size for migration assays. A549 cells were divided into two groups: the control group and the MnPB group. At 24 h after starvation, A549 cells of control group were resuspended in the serum-free RPMI 1640 medium and cells of MnPB group were resuspended in serum-free RPMI 1640 medium with MnPB NPs (400 μg/ml). Subsequently, the A549 cells were added to the upper chamber and the lower plate was filled with RPMI 1640 medium containing 10% FBS. After 24 h, A549 cells migrated to the membranes of the upper chamber were fixed with 4% paraformaldehyde and stained with crystal violet. Ultimately, images were obtained using a fluorescence microscope (Leica DMi8, Leica, Germany).

### Western Blot Analysis

A549 cells were grown in the 6-well plates and divided into the control group and the MnPB group. Protein harvesting and immunoblotting were performed as previously described (Fang et al., 2021). The primary antibodies included anti-MMP2 (Abcam, United States), anti-MMP9 (Abclonal, China) and anti-β-actin (Proteintech, China). And corresponding secondary antibodies were goat anti-rabbit IgG horseradish peroxidase (HRP) and goat anti-mouse IgG-HRP (Jackson Immunoresearch, United States).

### *In Vivo* Xenograft Assay

When the tumor volume reached the size of 7–9 mm, mice were randomly divided into four groups (*n* = 6 per group). The mice were treated as follows: 1) control; 2) PBS + NIR; 3) MnPB (2 mg/ml); 4) MnPB (2 mg/ml) + NIR. After 8 h of intravenous injection, the mice of group 2 and 4 were exposed to the irradiation (808 nm, 1 W/cm^2^) for 10 min and the change of temperature on tumor was recorded by a thermal imaging camera (FLIR A300). Meanwhile, tumor size and body weight of mice were measured ever 2 days. And the major organs were collected for histological analysis and the tumor samples were fixed and embedded for immunohistochemistry and immunofluorescence after 14 days.

### Ki67 Staining and TUNEL Assay

The tumor tissue specimens of each group were fixed with 4% paraformaldehyde, embedded in paraffin and sectioned. For Ki67 staining, the section slides were incubated by anti-Ki67 antibody (Abcam, United States) at 4°C overnight and then incubated for 1 h with secondary antibody at room temperature. Next, the sections were stained with diaminobenzidine (DAB) and hematoxylin respectively, dehydrated and mounted. And for TUNEL staining, the tissue blocks were incubated with TUNEL reaction mixture of TUNEL apoptosis detection kit (Servicebio, Wuhan, China) according to the manufacturer’s illustrations. And we used a microscope (Leica DMi8, Leica, Germany) to acquire images.

### *In Vivo* Magnetic Resonance Imaging

Tumor-bearing mice were received intratumoral injection of 200 μl MnPB NPs at the concentration of 2 mg/ml. Then the mice were scanned by a 3.0T MRI system before and 1 h after injection. In this way, the high-resolution T1-weighted MRI scan images were successfully acquired. The T1-weighted MRI parameters were as follows: pulse waiting time (TR) = 500 ms, echo time (TE) = 15 ms, slice thickness (ST) = 2.0 mm, field of view (FOV) = 60 mm × 60 mm.

### Statistical Analysis

All measurements were expressed as mean ± standard deviation (SD). And the data was analyzed by GraphPad Prism 7. Differences between groups were tested using the paired or unpaired two-tailed Student’s t-test. Besides, *p < 0.05, **p < 0.01, ***p < 0.001, ****p < 0.0001 were regarded to be significantly different.

## Results and Discussion

### Characterization and Properties of Manganese-Based Prussian Blue Nanoparticles

**FIGURE 1:**
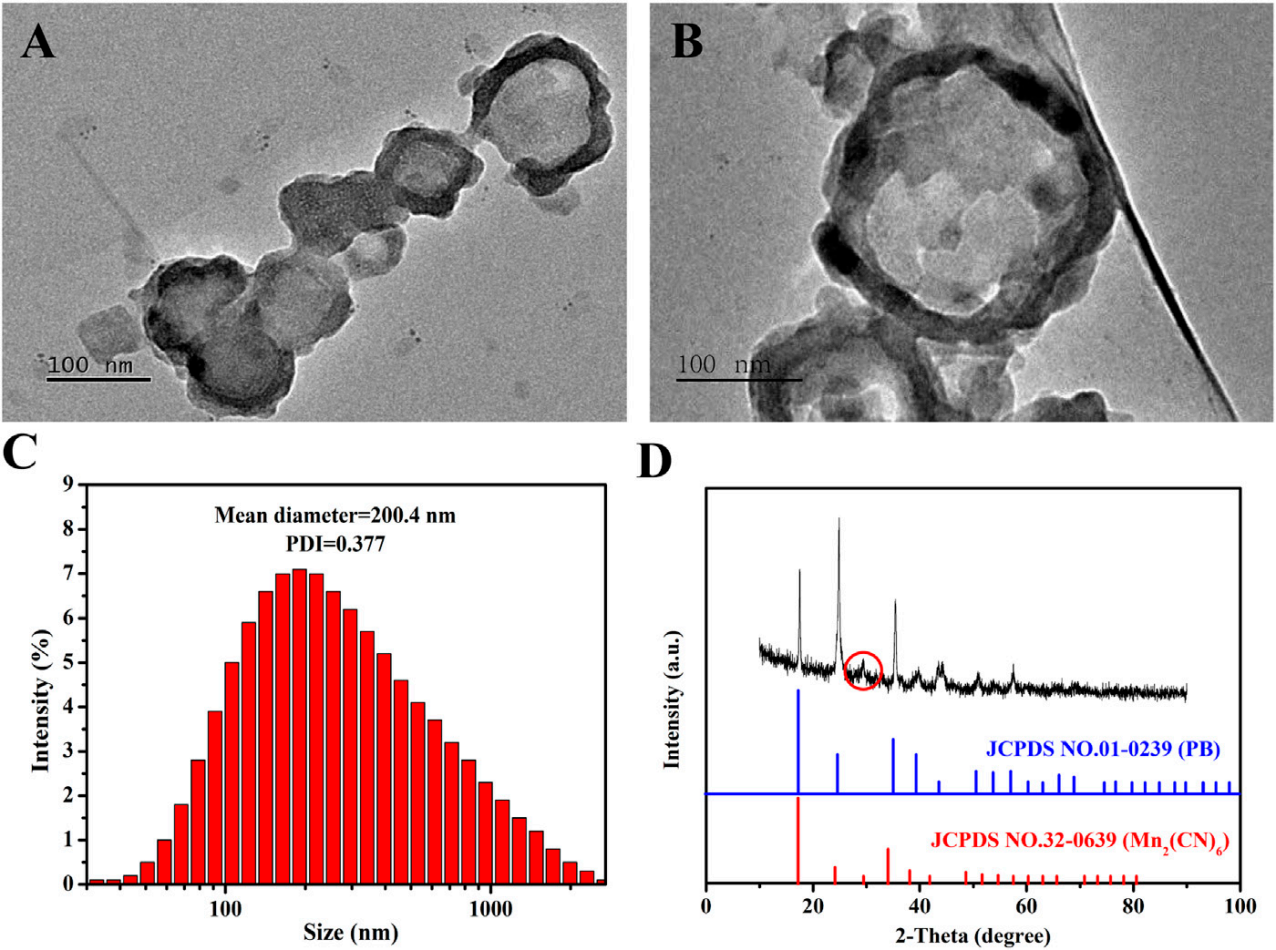
The characterization of MnPB NPs. **(A,B)** TEM micrographs of MnPB NPs. **(C)** The hydrodynamic size of MnPB NPs. And the zeta potential of MnPB NPs is −30.53 mV. **(D)** XRD patterns of as-synthesized MnPB NPs.

We synthesized the MnPB NPs using a conventional method, which substituted FeCl_3_ with MnCl_2_ to produce Mn-based PB. And the average size, shape and morphology of MnPB NPs were observed by TEM. The solid MnPB NPs were displayed in Figures 1A,B. And as we can see, the hydrodynamic size of MnPB NPs was measured to be 200.4 nm (Figures 1C), which was consistent to the size of TEM image. Then Figure 1D showed the XRD patterns of as-synthesized MnPB NPs, which revealed the characteristic peaks of PB and Mn^2+^, in accordance with the standard PB (JCPDS. 01-0239) and Mn_2_(CN)_6_ (JCPDS. 32-0639). The above results indicated the successful construction of MnPB NPs.

**FIGURE 2:**
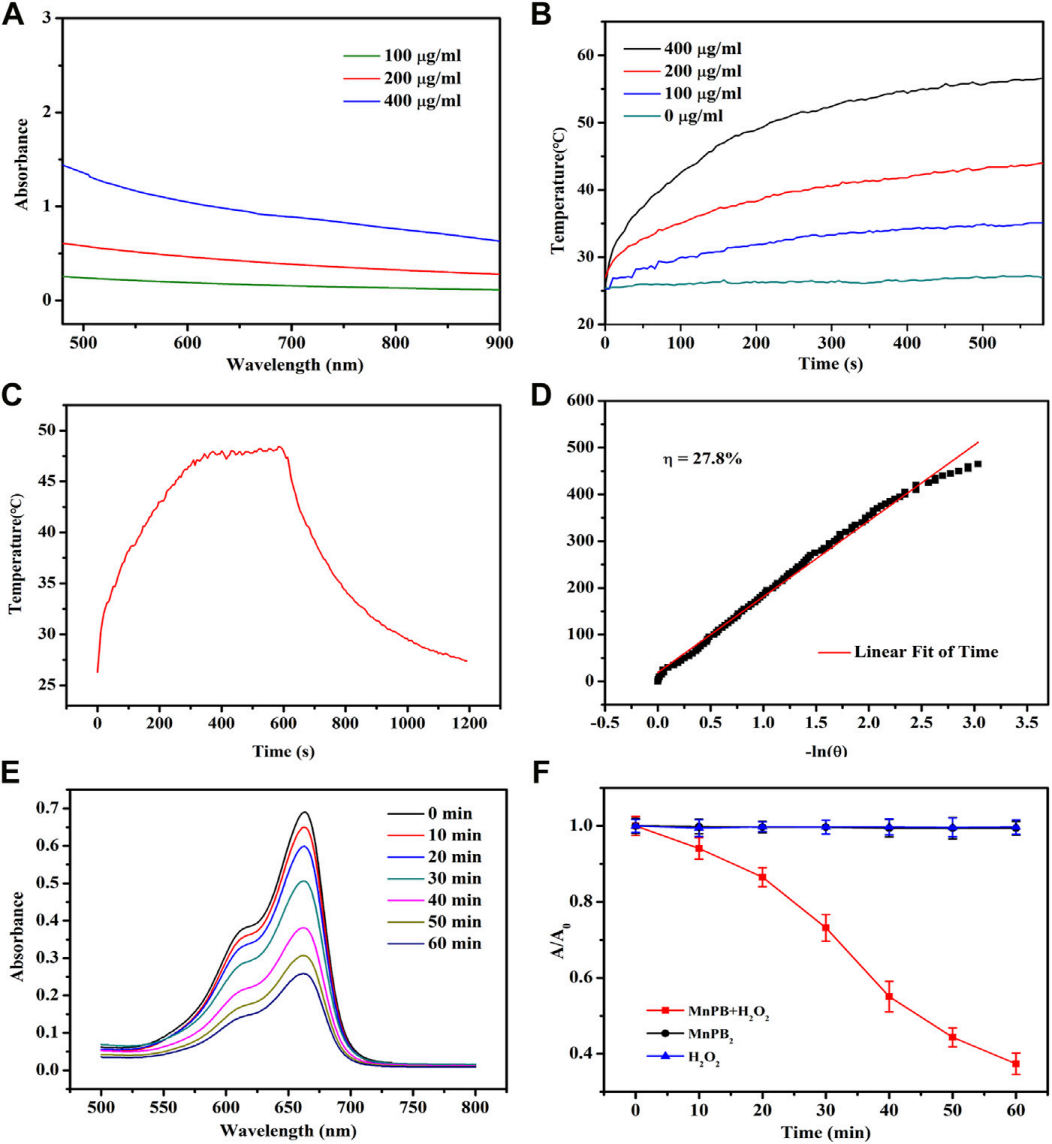
The photothermal properties of MnPB NPs. **(A)** UV−vis−NIR absorption spectra of MnPB NPs at varying concentrations. **(B)** Temperature profiles of MnPB NPs at various concentrations under NIR laser irradiation (808 nm, 1.0 W/cm^2^). **(C)** The heating-cooling curve of MnPB NPs at 200 μg/ml under NIR laser irradiation (808 nm, 1.0 W/cm^2^). **(D)** Red linear regression curve for natural cooling process. **(E)** Degradation of MB treated with MnPB NPs and H_2_O_2_ versus time. **(F)** Absorbance of MB at 664 nm in different groups.

To confirm the photothermal properties of MnPB NPs, UV−vis−NIR spectra of MnPB NPs with various concentrations were measured. As can be seen from Figure 2A, MnPB NPs exhibited strong and wide absorbance from visible wavelength to NIR wavelength ranges, illustrating the excellent light absorption ability of MnPB NPs. Then we further tested the photothermal performance of MnPB NPs at different concentrations under exposure of NIR light irradiation. At any given concentration of MnPB NPs, temperature rose steadily when the irradiation time increased (Figure 2B). With concentrations up until 400 μg/ml, the temperature rose above 50°C after 5 min of NIR light irradiation. It has been found that cells will die rapidly due to microvascular thrombosis and ischemia, as the temperature reaches above 45°C (Knavel and Brace, 2013). Thus, the photothermal ablation of MnPB NPs is highly sufficient to irreversibly ablate tumor cells. The photothermal conversion efficiency (ŋ) indicates the capability of converting light energy into thermal energy. The ŋ value of MnPB NPs was figured out to be 27.8%, which was higher than ŋ of PB NPs (only 16.02%) (Figures 2C,D) (Guan et al., 2021). These results suggested that MnPB NPs possessed excellent photothermal properties and were promising PTT agents for tumor treatment.

MB dye test is a simple procedure to evaluate hydroxyl radicals (•OH) formed in the Fenton’s reaction. And MB will turn from blue in color to colorless in the presence of •OH, which can be detected by a spectrophotometer (Satoh et al., 2007). As shown in the Figure 2E, MB displayed an absorbance peak at a wavelength of 664 nm, and the intensity decreased over time in MnPB NPs + H_2_O_2_ group, revealing the degradation of MB. And compared to the MnPB NPs group and H_2_O_2_ group, the absorbance measured at 664 nm in MnPB NPs + H_2_O_2_ group declined sharply, confirming that MnPB NPs could generate ROS effectively under the catalysis of H_2_O_2_ (Figure 2F). These observations demonstrated that the CDT of MnPB NPs had high efficiency and MnPB NPs might have powerful ability to inhibit the growth and metastasis of tumor.

### The Manganese-Based Prussian Blue Nanoparticles Inhibit the Growth of A549 Cells by Reactive Oxygen Species Upregulation and Near-Infrared Irradiation

**FIGURE 3:**
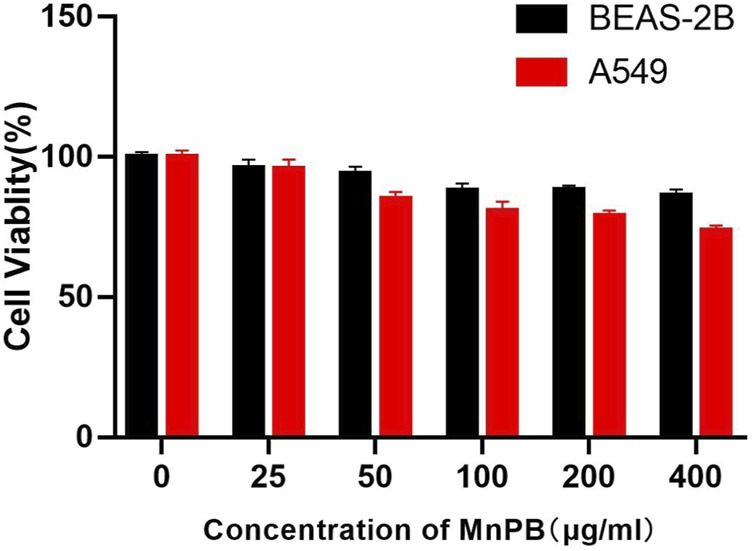
Cytotoxicity of MnPB NPs in A549 cells and BEAS-2B cells.

Biocompatibility of therapeutic agents is a critical feature for biomedical application. So the biocompatibility of MnPB NPs in normal bronchial epithelial cells and lung adenocarcinoma cells were firstly measured by CCK8 method. As Figure 3 shown, MnPB NPs exhibited no obvious cytotoxicity in both BEAS-2B and A549 cells at the maximum concentration of 400 μg/ml, which suggested excellent biocompatibility of MnPB NPs. Then we further evaluated the treatment effect of MnPB NPs with PTT and CDT in A549 cells.

**FIGURE 4:**
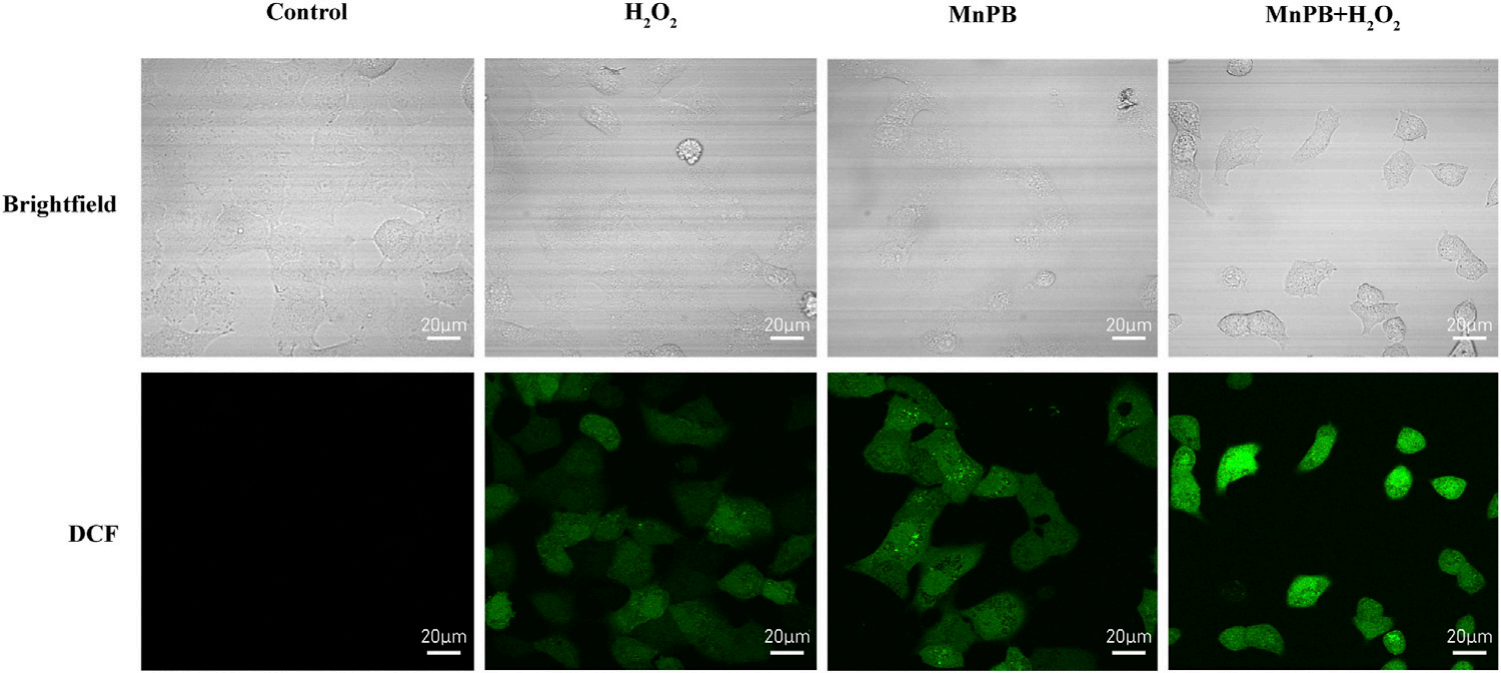
The MnPB NPs increase the intracellular ROS production in non-small lung cancer cells. Confocal images of intracellular ROS generation and corresponding brightfield images in A549 cells in different groups. Scale bar, 20 μm.

CDT is a new therapeutic approach for the treatment of tumor depending on Fenton and Fenton-like reactions. The Fenton reaction is defined as the production of highly oxidized •OH or O_2_ from H_2_O_2_ by catalyzing the transition metal ions or their compounds (Tang et al., 2019). To confirm the CDT effect of MnPB NPs *in vitro*, we used DCFH-DA probe to detect the ROS production (Figure 4; Supplementary Figure S1). The images displayed that, compared to the control group, DCF fluorescence intensity increased slightly when A549 cells were treated with H_2_O_2_ or MnPB NPs. And when treated with both H_2_O_2_ and MnPB NPs, most cells revealed intense green fluorescence signal. The findings suggested that MnPB NPs had a high efficiency of ROS generation through reacting with H_2_O_2_ in tumor microenvironment (TME) *via* Fenton and Fenton-like reactions. These reactions can be explained as follows:

$${\text{Fe}}^{2+}+{\text{H}}_{2}{\text{O}}_{2}\to {\text{Fe}}^{3+}+\cdot \text{OH}+{\text{OH}}^{-}$$ (1)

$${\text{Mn}}^{2+}+{\text{H}}_{2}{\text{O}}_{2}\to {\text{Mn}}^{2+}+\cdot \text{OH}+{\text{OH}}^{-}$$ (2)

**FIGURE 5:**
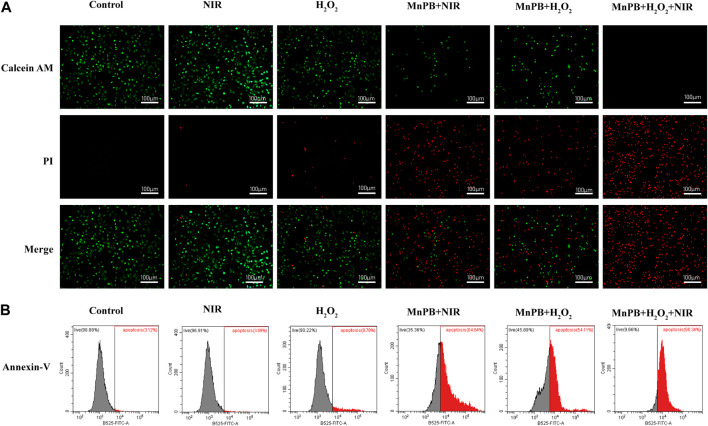
The MnPB NPs can induce cell apoptosis by ROS upregulation and NIR irradiation. **(A)** Confocal images of A549 cells stained with Calcein-AM and PI after different treatments. Scale bar, 100 μm. **(B)** Flow cytometry analysis of cell apoptosis with Annexin V-FITC staining.

Then based on the outstanding photothermal conversion performance of MnPB NPs, we applied two other methods to estimate the cell viability of A549 cells by combing PTT and CDT. First, we used Calcine-AM/PI test to detect the percentage of living and dead cells. Live cells were dyed with Calcein AM exhibiting green fluorescence, and dead cells were stained with PI showing red fluorescence. As shown in Figure 5A; Supplementary Figure S2, the green/red fluorescence intensity of A549 cells exposed under NIR irradiation had no significant difference, compared to the control group, indicating that cell viability was not affected by NIR irradiation. And H_2_O_2_ treatment increased the percentage of dead cells slightly. Besides, when A549 cells were treated with MnPB NPs with NIR light exposure or MnPB NPs with H_2_O_2_, the percentage of dead cells increased substantially, suggesting that the PTT and CDT of MnPB NPs had excellent performance respectively in A549 cells. And when A549 cells were treated with both PTT and CDT, we could only observe strong red fluorescence, revealing that the combination of PTT and CDT exerted potent efficacy to kill NSCLC cells.

Next, we used the Annexin V-FITC apoptosis assay to determine the apoptosis rate of A549 cells by flow cytometry. Apoptotic cells express phosphatidylserine (PS) on the outer layer of the cell membrane and can be recognized by Annexin-V specifically (Chen et al., 2008). As we can see in Figure 5B and Supplementary Figure S3, there was no obvious difference between the control group and the NIR group. But the rate of apoptosis increased to 9.78% in the H_2_O_2_ group. After treatment of MnPB NPs with NIR light exposure and MnPB NPs with H_2_O_2_, the rate of apoptosis obviously increased to 64.64% and 54.11%. Importantly, when cells were treated with MnPB NPs and exposed to both H_2_O_2_ and NIR irradiation, the percentage of apoptotic cells dramatically increased to 90.34%. The results were in good agreement with the observation of Calcine-AM/PI test, further demonstrating that PTT and CDT of MnPB NPs could act a strong synergy to inhibit growth of NSCLC cells.

### The Manganese-Based Prussian Blue Nanoparticles Inhibit the Migration of A549 Cells by Decreasing the Matrix Metalloproteinases Expression

**FIGURE 6:**
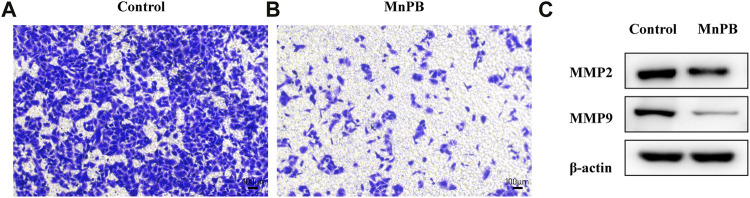
The MnPB NPs inhibit the metastasis of non-small lung cancer cells. **(A)** and **(B)** Migration capacity of A549 cells exposed to MnPB NPs. **(C)** MMP2 and MMP9 expression of A549 cells in different groups.

It is known that metastasis is a key feature of malignant tumors and major cause of death in cancer. Therefore, inhibition of tumor metastasis is extremely important in lung cancer treatment. Tumor metastasis is a complex multistep process related to cell migration, invasion, epithelial-to-mesenchymal transition (EMT), ECM degradation and intravascular circulation (Xu et al., 2014; Liu et al., 2021). Among these processes, the degradation of ECM is a critical step during the development of tumor metastasis (Eble and Niland, 2019). Here we utilized transwell assay to assess the inhibitory effect of MnPB NPs in A549 cells. It was obvious that the MnPB NPs significantly slowed down the migration of A549 cells (Figures 6A,B; Supplementary Figure S4).

It is proved that activity of matrix-degrading protease is important in the degradation of ECM, which plays an important role in invasion and migration of tumor. MMP2 and MMP9 reportedly degrade type IV collagen in the basement membrane, which is related to tumor metastasis (Wang et al., 2018). To further elucidate the inhibitory effects of MnPB NPs, we detected the activity of MMP2 and MMP9 in A549 cells. The MMP2 and MMP9 expressions were significantly attenuated by MnPB NPs, compared to the control group (Figure 6C; Supplementary Figure S5). To sum up, MnPB NPs can effectively inhibit the metastasis of A549 cells.

### The Manganese-Based Prussian Blue Nanoparticles Inhibit Xenografted Tumor Growth

**FIGURE 7:**
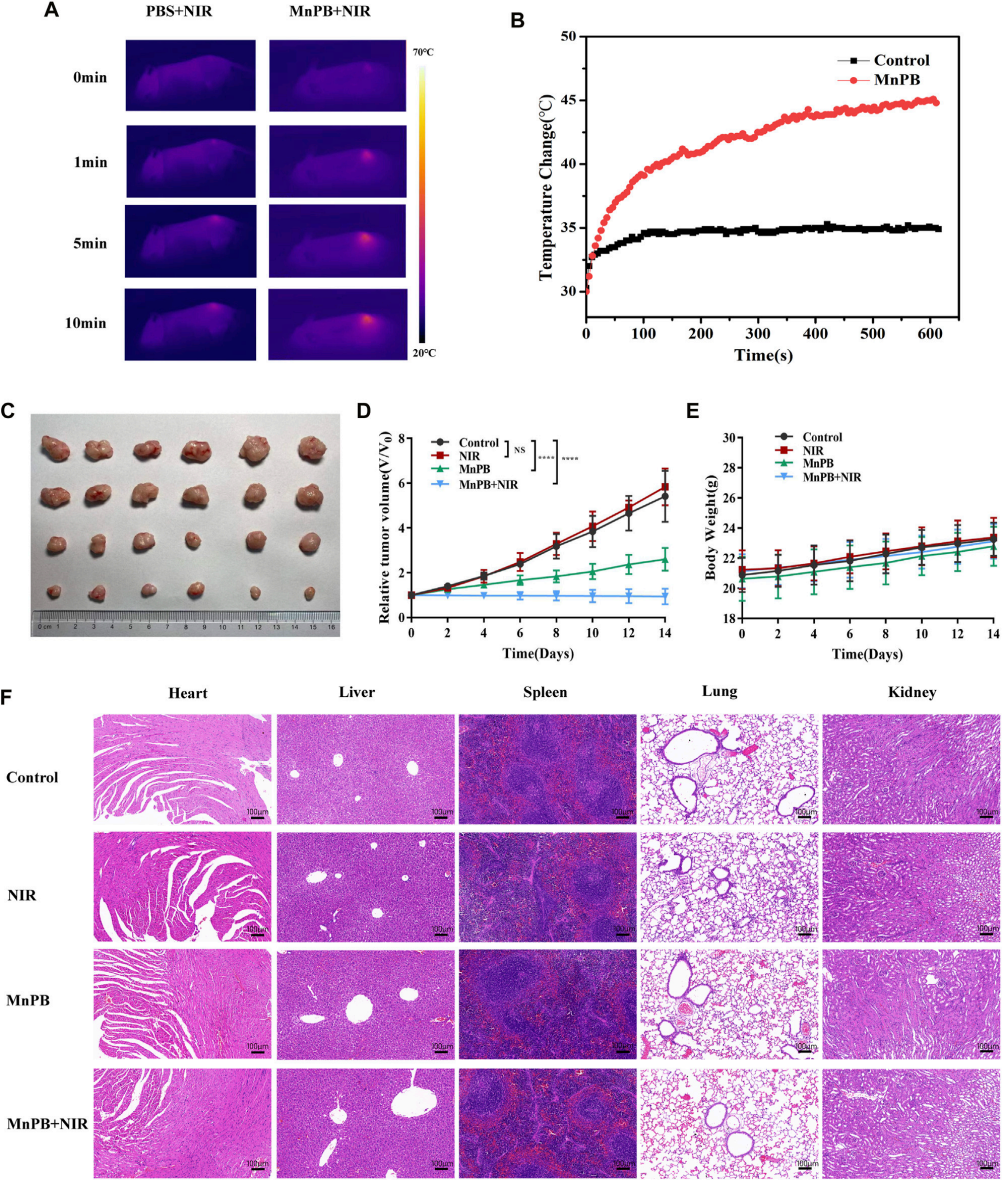
The MnPB NPs inhibit the growth of tumor xenografts in nude mice. **(A)** Pictures recorded by a thermal imaging camera during NIR irradiation. **(B)** Temperature curve of tumor areas in mice. **(C)** The tumor picture taken at day 14. **(D)** Relative tumor volume curve and **(E)** body weight curve of mice with various treatments. **(F)** H&E staining of main organs from mice in different groups. Scale bar, 100 μm.

According to the excellent therapeutic efficacy of *in vitro* studies presented above, we further assessed the *in vivo* performance of MnPB NPs. The tumor-bearing mice were randomly divided into four groups as follows (*n* = 6 per group): 1) control; 2) PBS + NIR; 3) MnPB NPs; 4) MnPB NPs + NIR. The mice of group 2, 3 and 4 were received 200 μl PBS or MnPB NPs intravenously. And the mice of group 2 and 4 were exposed under irradiation with an 808 nm NIR laser (1 W/cm^2^) for 10 min after 8 h of intravenous injection. During laser irradiation, we captured the change of tumor temperature by a thermal camera (Figures 7A,B). The temperature of tumor in group 2 only increased to ∼35°C, while the tumor temperature of group 4 rose rapidly above 45°C, which was high enough to induce tumor cell death. And the typical tumor pictures and the change in relative tumor volume (Figures 7C,D) demonstrated that only NIR had no influence to tumor growth, while only MnPB NPs could effectively inhibit the growth of tumor due to CDT effect. Surprisingly, treatment of MnPB NPs and NIR irradiation greatly contributed to the inhibition of tumor growth, which revealed that PTT and CDT of MnPB NPs have superior synergistic effect. Moreover, the body weight of mice in each group did not loss significantly during the treatment (Figure 7E). And we further examined the histology of main organs by H&E staining (Figure 7F). There were no appreciable abnormalities and pathological injury among the groups, indicating that MnPB NPs were biologically safe *in vivo*.

**FIGURE 8:**
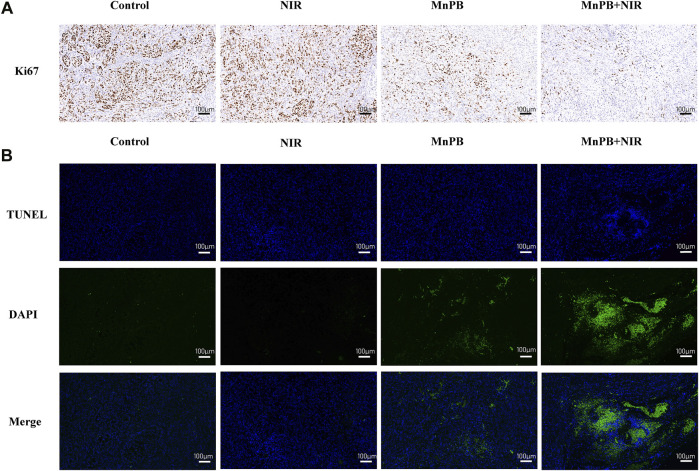
The MnPB NPs inhibit the growth of non-small lung cancer *in vivo*. **(A)** Ki67 staining and **(B)** TUNEL staining images of tumors. Scale bar, 100 μm.

We also used immunocytochemistry and immunofluorescence to assess cell proliferation and apoptosis of tumor tissue sections (Figures 8A,B; Supplementary Figures S6, S7). Compared to the control group, the expression of the proliferative marker Ki67 declined and cell apoptosis increased in the group 3 and 4. And resulting from synergistic therapeutic effect of CDT and PTT, most of the cells were apoptotic in group 4. These results clearly indicated that MnPB NPs with NIR irradiation led to severe cell damage and inhibited tumor growth efficiently *in vivo*.

### The Manganese-Based Prussian Blue Nanoparticles Serve as Great T1 Contrast Agents

**FIGURE 9:**
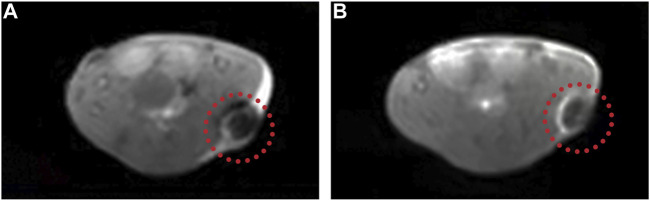
The MnPB NPs can be used as excellent T1 contrast agents. **(A)** Representative T1-weighted MRI images of A549 tumor-bearing mice before and **(B)** after intratumoral injection of MnPB NPs.

MRI is a widely used tool to diagnose clinical diseases with good soft tissue contrast and high spatial resolution (Yang et al., 2011; Liang et al., 2020). Nowadays, it is requisite to develop a multifunctional nanoplatform which includes noninvasive therapy methods and imaging technology (Zeng et al., 2019; Li X. et al., 2021). In our study, since MnPB NPs contained Mn^2+^, they could serve as T1 contrast agents. So we explored the T1-weighted imaging property of MnPB NPs *in vivo* (Figures 9A,B). The results showed that the MRI signal became brighter obviously after intratumoral injection of MnPB NPs, demonstrating that the MnPB NPs could act as effective T1 contrast agents.

## Conclusion

PB is well known as a safe photothermal agent in the treatment of tumor (Fu et al., 2012; Lu et al., 2020). In our study, we fabricated Mn-based PB nanocatalysts to acquire enhanced synergetic effect of PTT and CDT under the guidance of MRI. By the combination of PTT and CDT, the MnPB NPs induced cell death effectively of NSCLC *in vivo* and *in vitro*. And they had inhibitory effect on the metastasis of NSCLC cells *via* decreasing the expression of MMP2 and MMP9. Moreover, the MnPB NPs exhibited excellent T1-weighted imaging performance *in vivo*. Therefore, the results above confirmed that the MnPB NPs had the potential in treating NSCLC, and also could be used as excellent T1 contrast agents to diagnose NSCLC.

## Data Availability

The raw data supporting the conclusions of this article will be made available by the authors, without undue reservation.

## Abbreviations

CCK-8, cell counting kit-8; CDT, chemodynamic therapy; DCFH-DA, dichlorodihydrofluorescein diacetate; DLS, dynamic light scattering; ECM, extracellular matrix; EMT, epithelial-to-mesenchymal transition; FDA, Food and Drug Administration; HRP, horseradish peroxidase; H_2_O_2_, hydrogen peroxide; MB, methylene blue; MMP, matrix metalloproteinase; MRI, magnetic resonance imaging; NIR, near-infrared; NPs, nanoparticles; NSCLC, non-small cell lung cancer; PB, prussian blue; PBS, phosphate buffered saline; PI, propidium iodide; PS, phosphatidylserine; PTT, photothermal therapy; ROS, reactive oxygen species; TEM, transmission electron microscopy; XRD, X-ray powder diffraction.

## References

[B1] Altorki N. K. Markowitz G. J. Gao D. Port J. L. Saxena A. Stiles B. (2019). The Lung Microenvironment: an Important Regulator of Tumour Growth and Metastasis. Nat. Rev. Cancer 19, 9–31. 10.1038/s41568-018-0081-9 30532012 PMC6749995

[B2] Busquets M. A. Estelrich J. (2020). Prussian Blue Nanoparticles: Synthesis, Surface Modification, and Biomedical Applications. Drug Discov. Today 25, 1431–1443. 10.1016/j.drudis.2020.05.014 32492486

[B3] Cai X. Gao W. Ma M. Wu M. Zhang L. Zheng Y. (2015). A Prussian Blue-Based Core-Shell Hollow-Structured Mesoporous Nanoparticle as a Smart Theranostic Agent with Ultrahigh pH-Responsive Longitudinal Relaxivity. Adv. Mat. 27, 6382–6389. 10.1002/adma.201503381 26393889

[B4] Chen S. Cheng A.-C. Wang M.-S. Peng X. (2008). Detection of Apoptosis Induced by New Type Gosling Viral Enteritis Virus *In Vitro* through Fluorescein Annexin V-FITC/PI Double Labeling. World J. Gastroenterol. 14, 2174–2178. 10.3748/wjg.14.2174 18407590 PMC2703841

[B5] Dacarro G. Taglietti A. Pallavicini P. (2018). Prussian Blue Nanoparticles as a Versatile Photothermal Tool. Molecules 23, 1414. 10.3390/molecules23061414 PMC6099709 29891819

[B6] Dong Q.-z. Wang Y. Tang Z.-p. Fu L. Li Q.-c. Wang E.-d. (2013). Derlin-1 Is Overexpressed in Non-small Cell Lung Cancer and Promotes Cancer Cell Invasion via EGFR-ERK-Mediated Up-Regulation of MMP-2 and MMP-9. Am. J. Pathology 182, 954–964. 10.1016/j.ajpath.2012.11.019 23306155

[B7] Eble J. A. Niland S. (2019). The Extracellular Matrix in Tumor Progression and Metastasis. Clin. Exp. Metastasis 36, 171–198. 10.1007/s10585-019-09966-1 30972526

[B8] Fang D. Jin H. Huang X. Shi Y. Liu Z. Ben S. (2021). PPy@Fe_3_O_4_ Nanoparticles Inhibit Tumor Growth and Metastasis through Chemodynamic and Photothermal Therapy in Non-small Cell Lung Cancer. Front. Chem. 9, 789934. 10.3389/fchem.2021.789934 34820358 PMC8606671

[B9] Fu G. Liu W. Feng S. Yue X. (2012). Prussian Blue Nanoparticles Operate as a New Generation of Photothermal Ablation Agents for Cancer Therapy. Chem. Commun. 48, 11567–11569. 10.1039/c2cc36456e 23090583

[B10] Gao X. Wang Q. Cheng C. Lin S. Lin T. Liu C. (2020). The Application of Prussian Blue Nanoparticles in Tumor Diagnosis and Treatment. Sensors 20, 6905. 10.3390/s20236905 PMC7730465 33287186

[B11] Goldstein S. Meyerstein D. Czapski G. (1993). The Fenton Reagents. Free Radic. Biol. Med. 15, 435–445. 10.1016/0891-5849(93)90043-t 8225025

[B12] Gonzalez-Avila G. Sommer B. Mendoza-Posada D. A. Ramos C. Garcia-Hernandez A. A. Falfan-Valencia R. (2019). Matrix Metalloproteinases Participation in the Metastatic Process and Their Diagnostic and Therapeutic Applications in Cancer. Crit. Rev. Oncology/Hematology 137, 57–83. 10.1016/j.critrevonc.2019.02.010 31014516

[B13] Guan S. Liu X. Fu Y. Li C. Wang J. Mei Q. (2022). A Biodegradable “Nano-Donut” for Magnetic Resonance Imaging and Enhanced Chemo/photothermal/chemodynamic Therapy through Responsive Catalysis in Tumor Microenvironment. J. Colloid Interface Sci. 608, 344–354. 10.1016/j.jcis.2021.09.186 34626980

[B14] Han L. Sheng B. Zeng Q. Yao W. Jiang Q. (2020). Correlation between MMP2 Expression in Lung Cancer Tissues and Clinical Parameters: a Retrospective Clinical Analysis. BMC Pulm. Med. 20, 283. 10.1186/s12890-020-01317-1 33115469 PMC7594265

[B15] He T. Luo Y. Zhang Q. Men Z. Su T. Fan L. (2021). Hyalase-Mediated Cascade Degradation of a Matrix Barrier and Immune Cell Penetration by a Photothermal Microneedle for Efficient Anticancer Therapy. ACS Appl. Mat. Interfaces 13, 26790–26799. 10.1021/acsami.1c06725 34061496

[B16] Itoh T. Tanioka M. Matsuda H. Nishimoto H. Yoshioka T. Suzuki R. (1999). Experimental Metastasis Is Suppressed in MMP-9-Deficient Mice. Clin. Exp. Metastasis 17, 177–181. 10.1023/a:1006603723759 10411111

[B17] Knavel E. M. Brace C. L. (2013). Tumor Ablation: Common Modalities and General Practices. Tech. Vasc. Interventional Radiology 16, 192–200. 10.1053/j.tvir.2013.08.002 PMC4281168 24238374

[B18] Li J. Yu X. Jiang Y. He S. Zhang Y. Luo Y. (2021). Second Near‐Infrared Photothermal Semiconducting Polymer Nanoadjuvant for Enhanced Cancer Immunotherapy. Adv. Mat. 33, 2003458. 10.1002/adma.202003458 33325584

[B19] Li W. Jia M. Wang J. Lu J. Deng J. Tang J. (2019). Association of MMP9-1562C/T and MMP13-77A/G Polymorphisms with Non-small Cell Lung Cancer in Southern Chinese Population. Biomolecules 9, 107. 10.3390/biom9030107 PMC6468416 30889876

[B20] Li X. Lovell J. F. Yoon J. Chen X. (2020). Clinical Development and Potential of Photothermal and Photodynamic Therapies for Cancer. Nat. Rev. Clin. Oncol. 17, 657–674. 10.1038/s41571-020-0410-2 32699309

[B21] Li X. Sun H. Li H. Hu C. Luo Y. Shi X. (2021). Multi‐Responsive Biodegradable Cationic Nanogels for Highly Efficient Treatment of Tumors. Adv. Funct. Mater. 31, 2100227. 10.1002/adfm.202100227

[B22] Li Z. Zeng Y. Zhang D. Wu M. Wu L. Huang A. (2014). Glypican-3 Antibody Functionalized Prussian Blue Nanoparticles for Targeted MR Imaging and Photothermal Therapy of Hepatocellular Carcinoma. J. Mat. Chem. B 2, 3686–3696. 10.1039/c4tb00516c 32263805

[B23] Liang K. Li Z. Luo Y. Zhang Q. Yin F. Xu L. (2020). Intelligent Nanocomposites with Intrinsic Blood-Brain‐Barrier Crossing Ability Designed for Highly Specific MR Imaging and Sonodynamic Therapy of Glioblastoma. Small 16, 1906985. 10.1002/smll.201906985 32003089

[B24] Lim E.-K. Kim T. Paik S. Haam S. Huh Y.-M. Lee K. (2015). Nanomaterials for Theranostics: Recent Advances and Future Challenges. Chem. Rev. 115, 327–394. 10.1021/cr300213b 25423180

[B25] Lin L. Wang S. Deng H. Yang W. Rao L. Tian R. (2020). Endogenous Labile Iron Pool-Mediated Free Radical Generation for Cancer Chemodynamic Therapy. J. Am. Chem. Soc. 142, 15320–15330. 10.1021/jacs.0c05604 32820914

[B27] Liu Q.-L. Zhang Z. Wei X. Zhou Z.-G. (2021). Noncoding RNAs in Tumor Metastasis: Molecular and Clinical Perspectives. Cell. Mol. Life Sci. 78, 6823–6850. 10.1007/s00018-021-03929-0 34499209 PMC11073083

[B28] Liu X. Li B. Fu F. Xu K. Zou R. Wang Q. (2014). Facile Synthesis of Biocompatible Cysteine-Coated CuS Nanoparticles with High Photothermal Conversion Efficiency for Cancer Therapy. Dalton Trans. 43, 11709–11715. 10.1039/c4dt00424h 24950757

[B29] Lu L. Zhang C. Zou B. Wang Y. (2020). Hollow Prussian Blue Nanospheres for Photothermal/Chemo-Synergistic Therapy. Int. J. Nanomedicine 15, 5165–5177. 10.2147/IJN.S252505 32764943 PMC7373408

[B30] Manivasagan P. Joe A. Han H.-W. Thambi T. Selvaraj M. Chidambaram K. (2022). Recent Advances in Multifunctional Nanomaterials for Photothermal-Enhanced Fenton-based Chemodynamic Tumor Therapy. Mater. Today Bio 13, 100197. 10.1016/j.mtbio.2021.100197 PMC8753377 35036895

[B31] Odda A. H. Xu Y. Lin J. Wang G. Ullah N. Zeb A. (2019). Plasmonic MoO_3−x_ Nanoparticles Incorporated in Prussian Blue Frameworks Exhibit Highly Efficient Dual Photothermal/photodynamic Therapy. J. Mat. Chem. B 7, 2032–2042. 10.1039/c8tb03148g 32254807

[B32] Oser M. G. Niederst M. J. Sequist L. V. Engelman J. A. (2015). Transformation from Non-small-cell Lung Cancer to Small-Cell Lung Cancer: Molecular Drivers and Cells of Origin. Lancet Oncol. 16, e165–e172. 10.1016/S1470-2045(14)71180-5 25846096 PMC4470698

[B26] Paolillo M. Schinelli S. (2019). Extracellular Matrix Alterations in Metastatic Processes. Int. J. Mol. Sci. 20, E4947. 10.3390/ijms20194947 31591367 PMC6802000

[B33] Poudel B. Ki H.-H. Luyen B. T. T. Lee Y.-M. Kim Y.-H. Kim D.-K. (2016). Triticumoside Induces Apoptosis via Caspase-dependent Mitochondrial Pathway and Inhibits Migration through Downregulation of MMP2/9 in Human Lung Cancer Cells. Acta Biochim. Biophys. Sin. 48, 153–160. 10.1093/abbs/gmv124 26758192

[B34] Satoh A. Y. Trosko J. E. Masten S. J. (2007). Methylene Blue Dye Test for Rapid Qualitative Detection of Hydroxyl Radicals Formed in a Fenton's Reaction Aqueous Solution. Environ. Sci. Technol. 41, 2881–2887. 10.1021/es0617800 17533853

[B35] Shokouhimehr M. Soehnlen E. S. Hao J. Griswold M. Flask C. Fan X. (2010). Dual Purpose Prussian Blue Nanoparticles for Cellular Imaging and Drug Delivery: a New Generation of T1-Weighted MRI Contrast and Small Molecule Delivery Agents. J. Mat. Chem. 20, 5251. 10.1039/b923184f

[B36] Shou P. Yu Z. Wu Y. Feng Q. Zhou B. Xing J. (2020). Zn 2+ Doped Ultrasmall Prussian Blue Nanotheranostic Agent for Breast Cancer Photothermal Therapy under MR Imaging Guidance. Adv. Healthc. Mat. 9, 1900948. 10.1002/adhm.201900948 31746549

[B37] Siegel R. L. Miller K. D. Fuchs H. E. Jemal A. (2021). Cancer Statistics, 2021. CA A Cancer J. Clin. 71, 7–33. 10.3322/caac.21654 33433946

[B38] Sun H. Yu T. Li X. Lei Y. Li J. Wang X. (2021). Second Near-Infrared Photothermal-Amplified Immunotherapy Using Photoactivatable Composite Nanostimulators. J. Nanobiotechnol 19, 433. 10.1186/s12951-021-01197-5 PMC8686222 34930269

[B39] Tang Z. Liu Y. He M. Bu W. (2019). Chemodynamic Therapy: Tumour Microenvironment-Mediated Fenton and Fenton-like Reactions. Angew. Chem. Int. Ed. 58, 946–956. 10.1002/anie.201805664 30048028

[B40] Wang X. Fan L. Cheng L. Sun Y. Wang X. Zhong X. (2020a). Biodegradable Nickel Disulfide Nanozymes with GSH-Depleting Function for High-Efficiency Photothermal-Catalytic Antibacterial Therapy. iScience 23, 101281. 10.1016/j.isci.2020.101281 32622263 PMC7334425

[B41] Wang X. Yang B. She Y. Ye Y. (2018). The lncRNA TP73‐AS1 Promotes Ovarian Cancer Cell Proliferation and Metastasis via Modulation of MMP2 and MMP9. J. Cell Biochem. 119, 7790–7799. 10.1002/jcb.27158 29904939

[B42] Wang X. Zhong X. Liu Z. Cheng L. (2020b). Recent Progress of Chemodynamic Therapy-Induced Combination Cancer Therapy. Nano Today 35, 100946. 10.1016/j.nantod.2020.100946

[B43] Xu H. Hou Z. Zhang H. Kong H. Li X. Wang H. (2014). An Efficient Trojan Delivery of Tetrandrine by poly(N-Vinylpyrrolidone)-Block-Poly(ε-Caprolactone) (PVP-B-PCL) Nanoparticles Shows Enhanced Apoptotic Induction of Lung Cancer Cells and Inhibition of its Migration and Invasion. Int. J. Nanomedicine 9, 231–242. 10.2147/IJN.S55541 24403829 PMC3883593

[B44] Yang H. Zhuang Y. Sun Y. Dai A. Shi X. Wu D. (2011). Targeted Dual-Contrast T1- and T2-Weighted Magnetic Resonance Imaging of Tumors Using Multifunctional Gadolinium-Labeled Superparamagnetic Iron Oxide Nanoparticles. Biomaterials 32, 4584–4593. 10.1016/j.biomaterials.2011.03.018 21458063

[B45] Yang Z. Luo Y. Hu Y. Liang K. He G. Chen Q. (2021). Photothermo‐Promoted Nanocatalysis Combined with H_2_S‐Mediated Respiration Inhibition for Efficient Cancer Therapy. Adv. Funct. Mat. 31, 2007991. 10.1002/adfm.202007991

[B46] Zeng K. Xu Q. Ouyang J. Han Y. Sheng J. Wen M. (2019). Coordination Nanosheets of Phthalocyanine as Multifunctional Platform for Imaging-Guided Synergistic Therapy of Cancer. ACS Appl. Mat. Interfaces 11, 6840–6849. 10.1021/acsami.8b22008 30693749

[B47] Zhao H. Wang J. Li X. Li Y. Li C. Wang X. (2021). A Biocompatible Theranostic Agent Based on Stable Bismuth Nanoparticles for X-Ray Computed Tomography/magnetic Resonance Imaging-Guided Enhanced Chemo/photothermal/chemodynamic Therapy for Tumours. J. Colloid Interface Sci. 604, 80–90. 10.1016/j.jcis.2021.06.174 34265694

[B48] Zhao W. Yu X. Peng S. Luo Y. Li J. Lu L. (2021). Construction of Nanomaterials as Contrast Agents or Probes for Glioma Imaging. J. Nanobiotechnol 19, 125. 10.1186/s12951-021-00866-9 PMC8091158 33941206

[B49] Zheng Q. Dong H. Mo J. Zhang Y. Huang J. Ouyang S. (2021). A Novel STAT3 Inhibitor W2014-S Regresses Human Non-small Cell Lung Cancer Xenografts and Sensitizes EGFR-TKI Acquired Resistance. Theranostics 11, 824–840. 10.7150/thno.49600 33391507 PMC7738869

[B50] Zheng Z. Chen Q. Rong S. Dai R. Jia Z. Peng X. (2020). Two-stage Activated Nano-Truck Enhanced Specific Aggregation and Deep Delivery for Synergistic Tumor Ablation. Nanoscale 12, 15845–15856. 10.1039/d0nr03661g 32696787

