# Supplementary material for: Manganese-Based Prussian Blue Nanocatalysts Suppress Non-Small Cell Lung Cancer Growth and Metastasis via Photothermal and Chemodynamic Therapy
Source: Front Bioeng Biotechnol. 2022 Jun 22;10:939158. doi: 10.3389/fbioe.2022.939158 (PMC9257087; doi:10.3389/fbioe.2022.939158)
Supplement: Supplementary file 1 [file DataSheet1.docx]

Supplementary Material

# Supplementary Figures


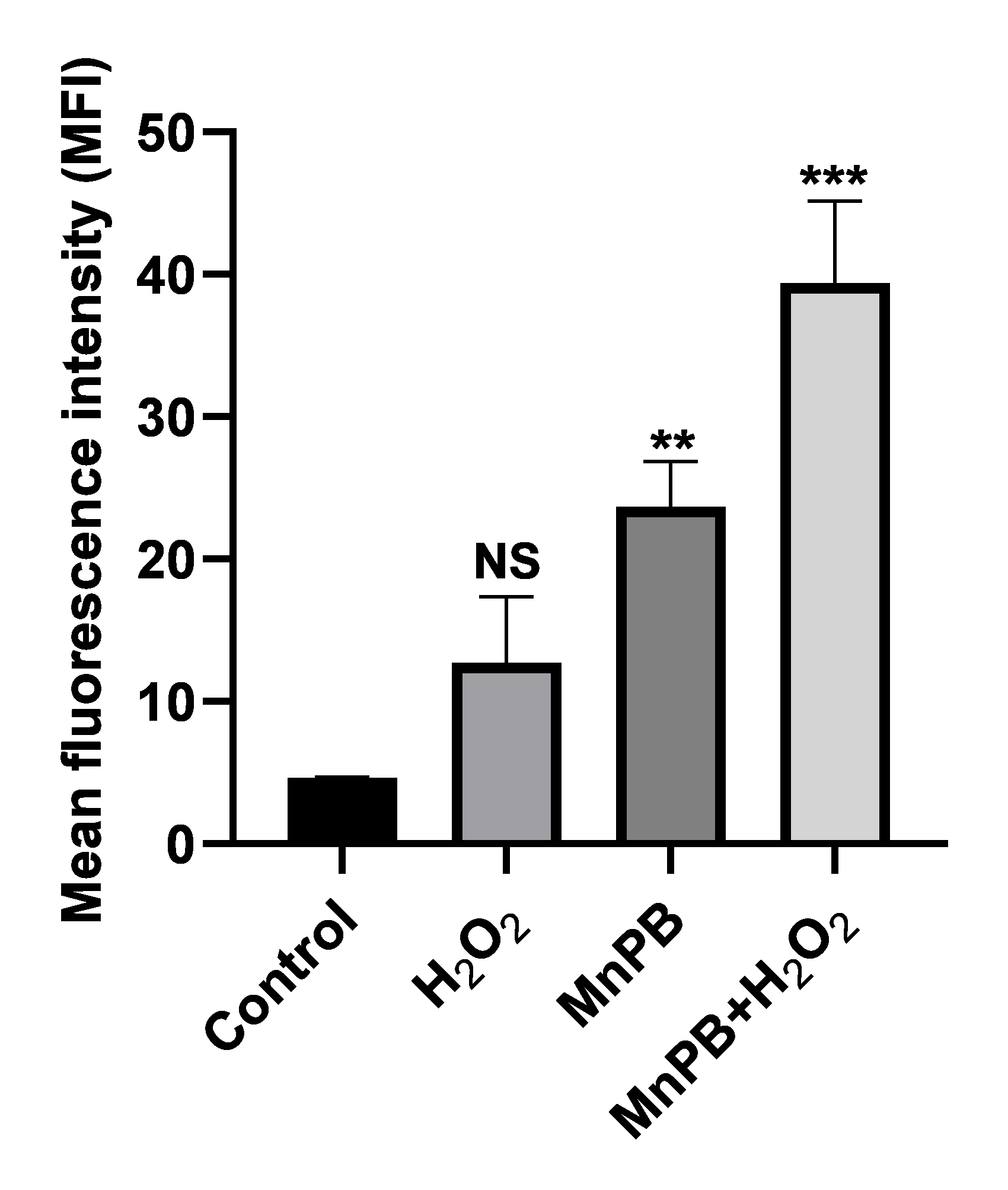


**Supplementary Figure 1.** Quantitative assessment of ROS generation according to mean fluorescence intensity (MFI). n=3, NS p > 0.05, ** p < 0.01 and *** p < 0.001.


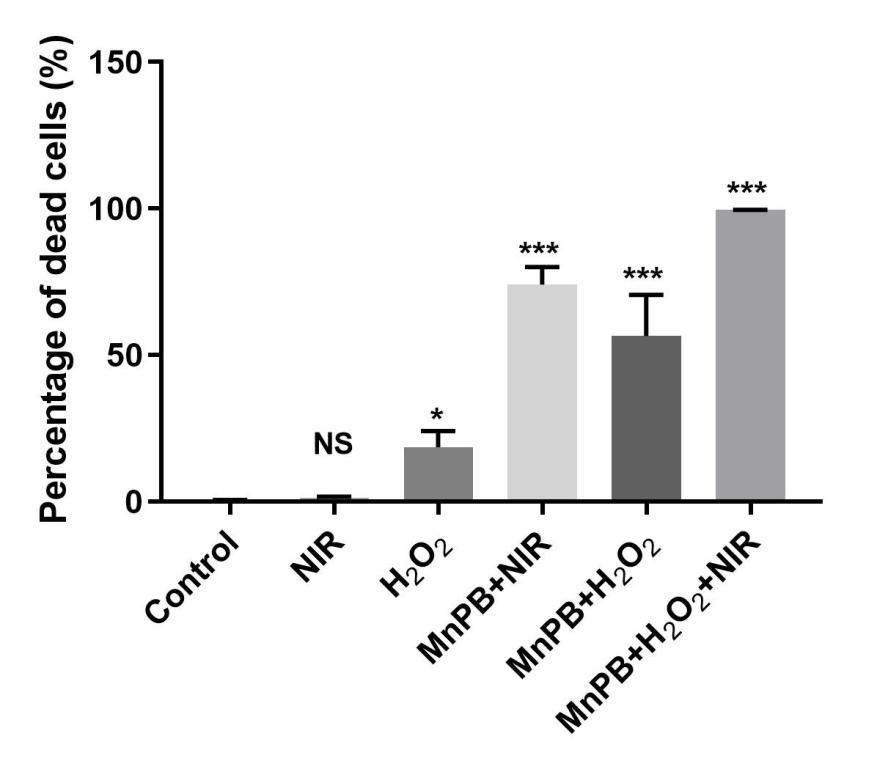


**Supplementary Figure 2.** The percentage of dead cells in Calcine-AM/PI test. n=3, NS p > 0.05, ** p < 0.01 and *** p < 0.001.


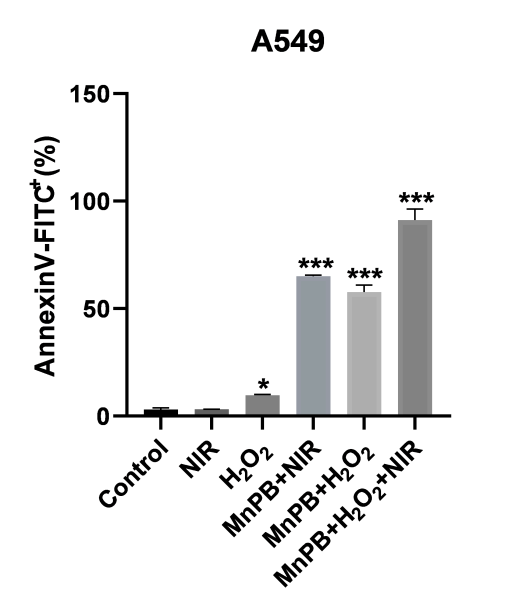


**Supplementary Figure 3.** The proportion of apoptosis of different groups in Annexin V-FITC apoptosis assay. n=3, NS p > 0.05, ** p < 0.01 and *** p < 0.001.


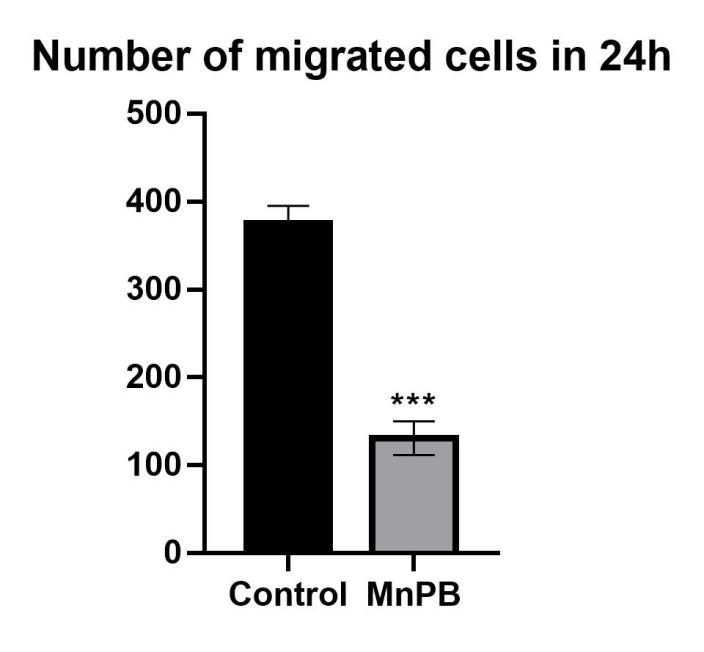


**Supplementary Figure 4.** The number of migrated cells of different groups in 24h. n=3, NS p > 0.05, ** p < 0.01 and *** p < 0.001.


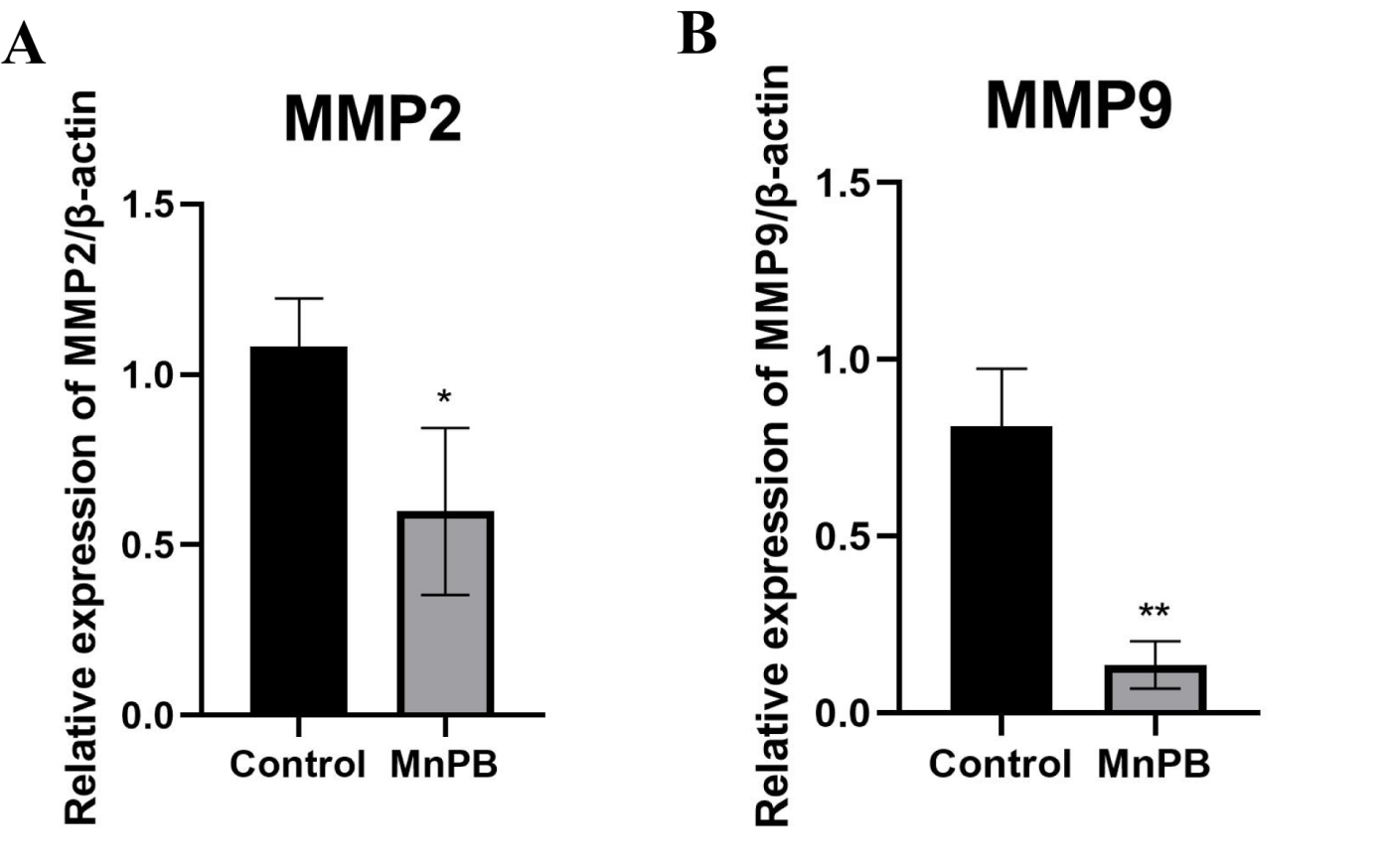


**Supplementary Figure 5.** (A)The relative protein expression of MMP2/β-actin in different groups. (B)The relative protein expression of MMP9/β-actin in different groups. n=3, NS p > 0.05, ** p < 0.01 and *** p < 0.001.


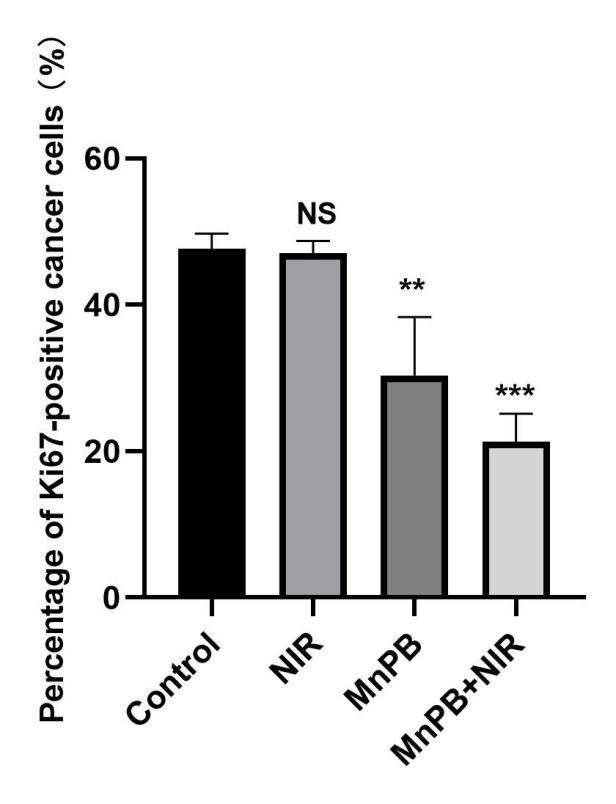


**Supplementary Figure 6.** The percentage of Ki67-positive cancer cells. n=3, NS p > 0.05, ** p < 0.01 and *** p < 0.001.


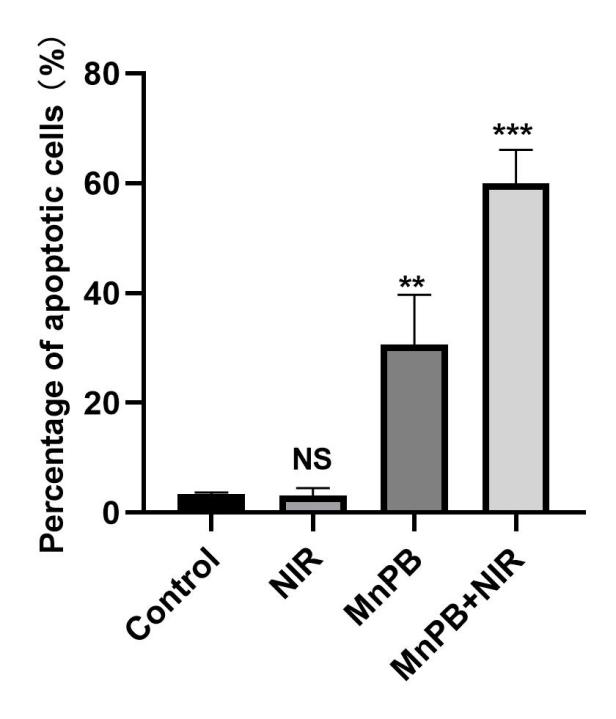


**Supplementary Figure 7.** The percentage of apoptotic cells. n=3, NS p > 0.05, ** p < 0.01 and *** p < 0.001.
